# Antimicrobial Resistance in *E. coli* Isolated from Chicken Cecum Samples and Factors Contributing to Antimicrobial Resistance in Nepal

**DOI:** 10.3390/tropicalmed7090249

**Published:** 2022-09-15

**Authors:** Pramesh Koju, Rajeev Shrestha, Abha Shrestha, Sudichhya Tamrakar, Anisha Rai, Priyanka Shrestha, Surendra Kumar Madhup, Nishan Katuwal, Archana Shrestha, Akina Shrestha, Sunaina Shrestha, Sandip K.C, Prashamsa Karki, Pooja Tamang, Pruthu Thekkur, Sony Shakya Shrestha

**Affiliations:** 1 Pharmacovigilance Unit, Dhulikhel Hospital, Dhulikhel 45210, Nepal; 2 Department of Public Health and Community Programs, Dhulikhel Hospital, Dhulikhel 45210, Nepal; 3 Department of Pharmacology, School of Medical Sciences, Kathmandu University, Dhulikhel 45210, Nepal; 4 Research and Development Division, Dhulikhel Hospital, Dhulikhel 45210, Nepal; 5 Department of Community Medicine, School of Medical Sciences, Kathmandu University, Dhulikhel 45210, Nepal; 6 World Health Emergencies Programme, WHO Country Office, Kathmandu 41825, Nepal; 7 Department of Microbiology, Dhulikhel Hospital, Dhulikhel 45210, Nepal; 8 Health Unit, Dhulikhel Municipality, Dhulikhel 45210, Nepal; 9 Centre for Operational Research, International Union Against Tuberculosis and Lung Disease (The Union), 75000 Paris, France

**Keywords:** animal, antimicrobial resistance, contributing factors, mixed-method study, multi-drug resistance

## Abstract

Microorganisms with antimicrobial resistance (AMR) are prevalent among humans and animals, and also found in the environment. Though organisms with AMR can spread to humans via food from animal sources, the burden of AMR in food-producing animals remains largely unknown. Thus, we assessed the resistance pattern among *Escherichia coli* isolated from chicken cecum samples and explored issues contributing to AMR in animals in the Dhulikhel Municipality of Nepal. We conducted a mixed-methods study, comprising a cross-sectional quantitative component, with collection of chicken cecal samples from slaughter houses/shops. In addition, a descriptive qualitative component was undertaken, with a focus group discussion and key informant interviews among stakeholders involved in animal husbandry. Of the 190 chicken cecum samples collected, 170 (89%) were subjected to culture and drug sensitivity testing, of which *E. coli* was isolated from 159 (94%) samples. Of the 159 isolates, 113 (71%) had resistance to ≥3 antimicrobial class. Resistance to tetracycline (86%) and ciprofloxacin (66%) were most prevalent. Overuse of antimicrobials, easy availability of antimicrobials, and lack of awareness among farmers about AMR were major issues contributing to AMR. The high prevalence of resistance among *E. coli* in chicken cecal samples calls for rational use of antimicrobials, educating farmers, and multi-sectoral coordination.

## 1. Introduction

Microorganisms with resistance to antimicrobials can affect people at any stage of life and also the animals (livestock) reared in the veterinary and agricultural sectors [1]. A study conducted in the United States showed that *Escherichia coli* (*E. coli*) isolates from livestock were more resistant than those from human clinical isolates [2]. Humans are exposed to antimicrobial-resistant microorganisms and their resistance genes are prevalent in animals, both via the food chain and through widespread release into the environment [3]. Pathogenic bacteria from sources such as livestock can interact with other bacteria, boosting the sharing of genes and genetic components that cause antibiotic resistance. These circumstances can cause non-pathogenic bacteria to develop into resistant reservoirs [4]. There is indisputable proof that food derived from a variety of animal sources contains large numbers of resistant bacteria and resistant genes [5]. A past study reported that chicken meat could be one of the potential causes of infection with multi-drug resistant (MDR) *E. coli* [6].

Considering the relatively low production cost and absence of cultural and religious restrictions on its consumption, poultry is one of the most widespread foods; chicken meat and eggs being the most common [7]. Antimicrobials are used, not only to treat disease in the poultry industry, but also to promote the growth of broiler chickens [8,9]. In Europe, use of avoparcin in food animals as a growth promoter had been linked with resistance to vancomycin, a last resort antimicrobial in human medicine [10]. Therefore, European countries have discontinued use of antimicrobials as growth promoters, but other countries in South America, Africa, and Asia still use it abundantly [11]. It has been found that approximately 80% of medically important antimicrobials are used as growth promoters in healthy animals, to fulfill the increasing demand for foods of animal origin [12,13]. The overall consumption of antimicrobials in livestock was estimated to have increased by 67% between 2010 and 2030 [14]. As a result of consumption and accumulation over time, there is higher chance of the development of multiple pathogens insensitive to medically important antimicrobials [15].

The World Health Organization’s (WHO) global action plan on antimicrobial resistance (AMR) emphasizes the “one health” approach, which recognizes the interconnections between humans, animals, and the environment as a single entity, to tackle resistance [16]. The one health approach provides important insights to plan and control the burden of AMR [17]. Systematic reviews on one health approaches have also shown associations between specific interventions targeting reductions in antibiotic use in food-producing animals and decreases in AMR in animals [18,19]. Knowledge on the burden of AMR and resistance patterns in isolates extracted from food-producing animals is imperative to designing targeted interventions to limit antibiotic use. The use of commensal intestinal *E. coli* as a marker for the presence of resistance in bacterial flora is a critical component of AMR surveillance programs in both food-producing and wild animals [20]. The chicken gut micro biota constitutes a major source of antibiotic resistance genes that encode several drug efflux pumps, leading to resistance to fluoroquinolones and tetracyclines [21].

In Nepal, the use of antimicrobials has increased in recent times, in order to decrease the morbidity and mortality of chickens [22,23]. A study conducted in Nepal under the Global Antibiotics Resistance Partnership (GARP) has shown that 46% of veterinary drugs were sold under self-prescription and about 12% on farmer’s demand [23]. Surveillance of animal pathogens commenced in 2011, with a collaboration between the National Public Health Laboratory and various veterinary laboratories. The Ministry of Health and Population, Nepal, attempted to address such issues with National Antibiotics Treatment Guidelines in 2014 [24]. There is no clear regulation for control of the use of antimicrobials in animals for human consumption.

Very few studies have assessed AMR in the poultry sector in Nepal. A study conducted on bacteriological quality of poultry meat in Nepal showed that various bacteria, such as *E. coli*, *Staphylococcus,* and *Klebsiella* showed higher resistance to commonly found antibiotics on the market, such as amoxicillin and tetracyclines [25]. While in other study, *E. coli* was found in 76.1% of poultry meat samples, and resistance to tetracycline was highest (87.7%) and lowest for ceftriaxone (1%) [26]. However, there is no published literature on the pattern of AMR in isolates from chicken cecal samples in Nepal. Cecal samples provide a better insight into the resistance pattern in the microbiota of the chicken and also are not prone to cross-contamination from the environment in the slaughter house.

Moreover, the previously conducted studies looked into the resistance pattern of various organisms, but there are no studies that identified the possible issues contributing to AMR in animals. Therefore, we aimed to estimate the prevalence of *E. coli* isolates, describe the resistance pattern, and assess the MDR from the *E. coli* isolated from chicken cecal samples. In addition, we aimed to explore the possible issues contributing to AMR in animals, in selected wards of Dhulikhel municipality.

## 2. Materials and Methods

### 2.1. Study Design

We adopted a concurrent mixed-methods study design, with quantitative and qualitative components. For the quantitative component, we conducted a cross-sectional study to determine the resistance pattern of *E. coli* in chicken cecal samples. For the qualitative component, we carried out a descriptive study using focus group discussions (FGDs) and key informant interviews (KIIs), to identify the issues contributing to AMR in animals.

### 2.2. Study Setting

Nepal falls under the subtropical region of the world. The climate, flora, and fauna vary in different regions [27]. Nepal is geographically divided into 7 provinces, 14 zones, and 77 districts. The majority of village farmers rely on agriculture and livestock farming for a living [28]. The livestock sector alone contributes about 11.5% of the gross domestic product (GDP) and 25.7% of the agricultural GDP (AGDP) [29]. There has been an increase in meat production by 24% in the last 10 years, mainly influenced by chicken meat. The tremendous growth in the poultry population has resulted in the independent contribution of the poultry sector to national GDP of about 4% [30].

This study was carried out in the Dhulikhel municipality of Kavrepalanchowk district, Nepal. We collected chicken cecal samples from all the slaughterhouses/chicken shops within two selected wards: rural and semi-urban (Ward 2 and Ward 6) out of the 12 wards in the municipality. There are only a few animal clinics in Dhulikhel municipality and 20 to 25 animal-based food retailers (registered and unregistered) in Dhulikhel municipality.

### 2.3. Study Population and Sampling

#### 2.3.1. Quantitative

The sample size was calculated using $Z_{\left(1-\alpha /2\right)}$ as a standard normal variate (1.96 at 5% type I error (*p* < 0.05), assuming 50% prevalence of MDR among the *E. coli* isolates with 15% precision, 10% wastage of collected sample, and 95% confidence interval. The minimum sample size for the study was 170 chicken cecal samples. The sample size was calculated using the following formula:

(1) $$Sample\;size=\frac{Z_{\left(1-\alpha /2\right)}{}^{2}p\left(1-p\right)}{d^{2}}$$

where $Z_{\left(1-\alpha /2\right)}$ is a standard normal variate, *p* is the expected proportion in a population, and *d* is the absolute error or precision.

We conveniently chose two wards (one rural and one semi-urban) where most of the people reside and consume meat and obtained informed consent from the owners of all the available slaughterhouses/chicken shops. The samples were drawn proportionately from the selected shops of both wards. The slaughterhouses where the owner did not give consent were excluded. In addition, the samples from chickens that had been treated with any medication or antimicrobials and those that were apparently sick before slaughtering were excluded from the study. A trained sample collector collected the cecal samples of chickens right after the slaughtering, to avoid potential contamination.

#### 2.3.2. Qualitative

In order to identify issues contributing to AMR in the animal population, we obtained a list of pharmacies, veterinarians, food vendors, health coordinators, and community members from Dhulikhel municipality. The participants for FGDs and KIIs were selected purposely, to include those whose representation had a key role in determining AMR. All the participants were above 18 years of age, currently residing in Dhulikhel, and also gave consent to participate in the study. Three FGDs (with 12 participants in each group) and 22 KIIs were conducted to obtain meaningful insights.

### 2.4. Study Variables, Sources, and Data Collection

#### 2.4.1. Quantitative

We collected cecal samples from chickens after slaughtering and transported them to the microbiology laboratory in a biohazard box (temp 2–8 °C). We also collected information regarding the types of meats being sold in the shop, the source of water used in the shop, and also whether a slaughter house was attached to the shop.

The cecal samples were cleaned with normal saline and each sample was given a unique identification number. A small amount of the mucosal scraping of the saline cleaned cecal sample was inoculated in MacConkey agar and incubated at 37 °C for 24 h [4]. Further sub-culture of the lactose fermenting colonies was done in MacConkey Agar [31]. For the confirmation of *E. coli*, Gram-staining and a number of biochemical tests, including oxidase, catalase, citrate utilization, urease, sulphur indole motility (SIM), and triple sugar iron (TSI) tests were carried out [32]. Different isolates of *E. coli* were identified based on their colony morphology, motility, and antimicrobial susceptibility testing (AST) pattern. The Kirby–Bauer disc diffusion method was used for AST, following the Clinical and Laboratory Standard Institute (CLSI) guidelines [33]. Antimicrobial discs were placed on the confluent lawn of the microbial suspension of *E. coli* (0.5 MacFarland Standard) on Mueller Hinton Agar [34]. After incubation of the plates for 16–18 h, the zone of inhibition (mm) was observed. For each tested antibiotic, the decision regarding sensitive, intermediate, and resistant was made in accordance with the manufacturer’s instructions [35]. We tested for cefotaxime, ciprofloxacin, ampicillin, tetracycline, chloramphenicol, gentamicin, and cotrimoxazole. Isolates which showed resistance to more than or equal to three classes of antimicrobials were considered MDR isolates [36].

#### 2.4.2. Qualitative

We conducted FGDs and KIIs among different study groups, as these methods provide a wide range of responses to open ended questions [37]. This method has an important role in clarifying the values, language, and meanings attributed to people who play different roles in organizations and communities [38]. We prepared different guides, in order to conduct FGDs and KIIs among the participants. These guides were pretested. Prior to data collection, a workshop was conducted to build the capacity of research assistants. A standard script was followed in a telephone call and verbal consent was obtained. Informed consent was obtained from the participants before data collection and audio recording. Participation in the study was completely voluntary. The FGDs and KIIs were conducted by trained researchers in the Nepali language. The interviews took about 35 min whereas the FGDs continued for about 60 to 90 min. We recorded audio and also took notes, with the consent of the participants. All information has been kept confidential.

### 2.5. Data Analysis

We entered the collected data in Epidata software v3.1 (EpiData Association, Odense, Denmark). The research team supervised the data entry, data cleaning, and data coding, which was cross validated by the principal investigator. Data were analyzed using Stata software v12.1 (Stata Corp, College Station, TX, USA). Laboratory results with demographic data were entered in excel, and resistance patterns were shown as frequencies and proportion.

A unique code was given to participants from the FGDs and KIIs, in order to maintain confidentiality. The audio was transcribed and translated from FGDs and KIIs into English. A codebook was developed based on the questions asked during the interviews and group discussions. Manual content analysis was conducted to deduce codes, and the codes were entered in Microsoft Excel. Thematic analysis was done using the codes, and verbatim quotes relevant to the codes were presented. Several codes which were related to the issues contributing to AMR were further grouped into similar categories.

## 3. Results

### 3.1. Quantitative Findings

**Figure 1:**
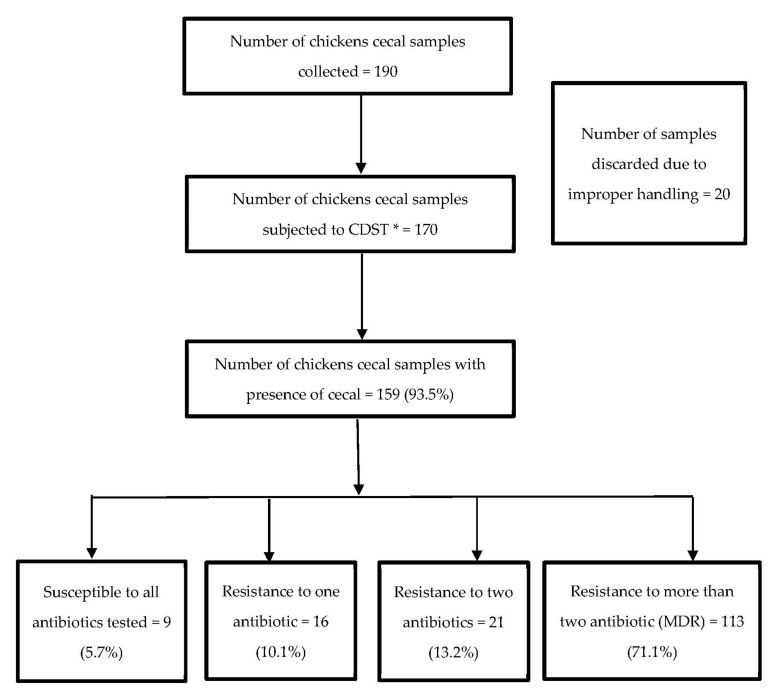
Flowchart depicting the pattern of *E. coli* in chicken cecal samples and resistance to antimicrobials in the Dhulikhel municipality of Nepal from September to December 2021. * Culture and drug sensitivity test.

A total of 190 chicken cecal samples were collected from September 2021 to December 2021 for the study. Of the total, 20 (10.5%) samples had to be discarded due to inappropriate transportation methods, leading to the chance of cross-contamination between samples. Of the 170 samples processed, *E. coli* was isolated from 159 (93.5%) cecal samples. Among those samples, 113 (71.1%) isolates were MDR (resistant to ≥3 classes of antimicrobials) (Figure 1).

The presence of *E. coli* (45, 93.8%) and the prevalence of MDR (34, 75.6%) was more common in Ward 2. The number of *E. coli* isolated was more in the samples collected from shops that sold only chicken (47, 94.0%), but the *E. coli* isolates from samples collected from shops that sold other meat along with chicken (88, 78.6%) reported a greater MDR pattern. The presence of *E. coli* (45, 93.8%) and MDR pattern (34, 75.6%) was higher in the samples from shops that brought meat samples from the slaughter house (Table 1).

Among the 159 cecal samples, six samples had two isolates of *E. coli*. Of the 165 isolates, more than 50% isolates were resistant to tetracycline (86%), ciprofloxacin (66.1%), ampicillin (60.0%), and cotrimoxazole (50.9%). Only 12 (7.3%) out of 165 isolates were resistant to cefotaxime (Table 2).

### 3.2. Qualitative Findings

**Figure 2:**
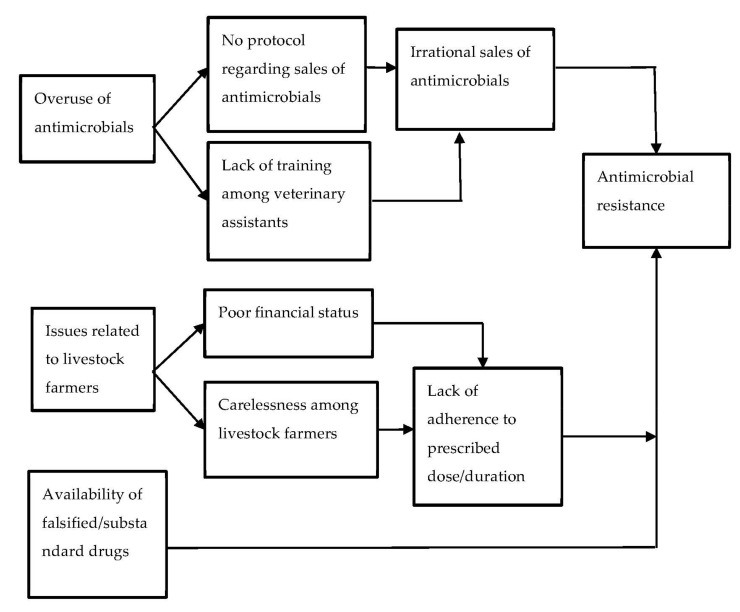
Issues contributing to AMR in the animal population as perceived by the ward chairperson, policymakers, and veterinary assistants of the Dhulikhel municipality, 2021.

In this study, three sub-themes were identified as the major drivers for AMR in the animal population (Figure 2). The drivers were further classified into categories based on the findings.

#### 3.2.1. Overuse of Antimicrobials

From the interviews and discussions, it was evident that antimicrobials were being overused, not only in humans, but also in animals. A ward chairperson during the interview revealed that animal husbandry centers use various antimicrobials to treat animals.

“Our pharmacy, hospital is selling antimicrobials, also the pesticides shop, animal husbandry centers are using antimicrobials a lot.”

(WC-6)

According to the vet assistants, overuse of antimicrobials was mostly prevalent in chickens, but also common in cows.

“Yes, antimicrobials are overused in broiler chickens, but also common in cows during mastitis.”

(V001)

Most of the food vendors were unaware of the use of antimicrobials in animals, as they were not involved in rearing the animals. Some of the reasons for overuse of antimicrobials in the animals mentioned during the interviews are highlighted below:

1. No guidelines regarding the sale of antimicrobials

Most of the participants mentioned that a lack of guidelines on sales of antimicrobials has led to irrational selling. This driver for AMR can lead to prescription of the wrong type and dosage of medicine by an unqualified practitioner. One of the district health office representatives said that grocery stores in rural areas sell medicine both for humans and animals.

“Especially in rural areas, the same store has human medicine, veterinary medicine, pesticides, and grocery items, there are no standard guidelines against such practices. Hence, we as a consumer should also be aware, the seller should also have awareness and medicines should be available only in pharmacies. In order to do so, laws and regulations should be prepared and follow up should be done at local level.”

(K001)

2. Lack of training among veterinary personnel

The majority of the ward chairpersons perceived that antimicrobials are being overused in animal husbandry, as the ones prescribing the antimicrobials are not trained. Such action leads to unnecessary use of antimicrobials in animals, in turn leading to AMR. One of them said,

“We have 4 veterinary clinics in our ward, most of the staff are not well trained, and that’s why there is overuse of antimicrobials. There is overuse but we don’t have the exact data.”

(WC-12)

3. Irrational sales of antimicrobials

One of the vet assistants felt that the rules and regulations were not enough to control irrational sales of antimicrobials and there was no effective implementation. He said that

“According to law, we can only sell ‘ga’ categories of over the counter (OTC) drugs. We are not allowed to sell ‘ka’ and ‘Kha’ categories of drugs without prescription. Everything is listed as rules but no one follows it.”

(V001)

#### 3.2.2. Issues Related to Livestock Farmers

1. Poor financial status

It was evident from the interviews with veterinary assistants that feeding medicine to an animal is not an easy task. Moreover, they frequently receive requests from farmers to dispense antimicrobials for shorter durations, which is because farmers are not able to afford the full course of antimicrobials.

A vet assistant explaining the same said,

“They don’t agree, if we recommend that they be fed for three days, they insist on purchasing for one day or ask us for half a dose of medicine. This is one of the main problems raised due to poor financial conditions.”

(V002)

Another vet assistant added,

“At first it’s due to lack of education …and then it’s the poor economic condition that needs to be addressed. The doctor recommends medicine for 3 days, but they request to dispense it only for 2 days so we send them incomplete doses.”

(V001)

2. Carelessness among the livestock farmers

Veterinary medicines are dispensed for a shorter duration than should be prescribed. The reason for such practice is due to the farmers not feeding the prescribed medicine for the recommended duration. A short duration prescription encourages the farmers to make visits for a refill after the completion of initial doses.

“Initially, we give medicine only for 3 days, not for 5 days. If we dispense medicine for 5 or 7 days, then the farmer might not feed full course. If it is to be fed for 5 days, he might give only one dose and forget. But if we dispense medicine for 3 days, he will return back for follow-up once the medicine is over.”

(V001)

The probable cause behind this is the difficulty in feeding the animals:

“It’s very difficult to feed medicine to cows and buffaloes for 5 or 7 days, feeding them is not an easy task.”

(V001)

#### 3.2.3. Availability of Falsified/Substandard Drugs

The interviews revealed the availability of substandard drugs, leading to the use of substandard drugs among animals more than in humans. As most of the farmers are illiterate, they may use such substandard drugs unintentionally. This has the potential to cause AMR.

“Usually most of the farmers are illiterate, they can’t even find out if it is expired or not, they will buy and use the medicines from medical stores on the basis of trust.”

(WC-12)

“The use of such drugs could be at higher rates than in humans.”

(WC-6)

## 4. Discussion

In our study, *E. coli* was isolated from more than 90% of the chicken cecal samples. Resistance to ≥3 classes of antimicrobials (MDR) was found in about seven out of ten *E. coli* isolates. The highest resistance was observed against tetracycline, followed by ciprofloxacin, ampicillin, and cotrimoxazole. The qualitative exploration showed that overuse of antimicrobials, easy availability of the falsified/substandard drugs, poor financial status, and lack of awareness about AMR among farmers were the major issues contributing to AMR in chickens.

A World Bank report in 2017 estimated that, by 2050, global livestock production will fall by 3% to 8% each year due to AMR. There may be a 11% loss in the livestock production, with the highest decline expected in low-income countries due to AMR, resulting in economic and development consequences [39]. Absence of effective implementation of veterinary drug use and coordination between authorities has resulted in an unorganized and haphazard veterinary market. This study has demonstrated the high prevalence of MDR *E. coli* in chicken cecal samples and has also identified issues contributing to AMR that require action.

The community-based mixed method design adopted for this study was its main strength, as it allowed us to estimate the resistance pattern and identify the issues contributing to the resistance. We also adhered to the COREQ and STROBE guidelines in reporting qualitative and quantitative findings, respectively. This study is subject to a few limitations. First, the study was only conducted in two wards of Dhulikhel municipality, and therefore the findings cannot be generalized to the whole country. Second, we were unable to carry out molecular characterization of the *E. coli* isolates in commensal or pathogenic organisms. Third, we assessed the resistance to a only limited number of antibiotics in this study. Lastly, the age of the chickens could not be determined in this study, as the slaughterhouses contained chickens from different batches bought at different times. The prevalence (94%) of *E. coli* in the chicken cecal samples found in the study mirrors the findings of a meta-analysis of nine different studies conducted in South Asia, which reported a *E. coli* prevalence of 84% in poultry [40]. Individual studies conducted elsewhere also reported the prevalence of *E. coli* to be more than 70% [26,41]. Similarly to this study, previous studies from the South Asia region reported that more than 70% of the *E. coli* were resistant to streptomycin, enrofloxacin, and sulphonamides, and almost 90% in the case of tetracycline [40]. Studies from Bangladesh and China reported high resistance against ampicillin and tetracycline among *E. coli* isolates from poultry samples [42,43]. Similarly, in Pakistan, *E. coli* isolated from chicken meat, chicken fecal, and respiratory secretion specimens showed more resistance to co-trimoxazole, chloramphenicol, and moxifloxacin than to other drugs [44]. In the study conducted in China, 89.20% of tested *E. coli* isolates from chicken fecal samples showed multi-drug resistance [42]. The antibiogram profile of *E. coli* isolates from broiler chickens in Chitwan, Nepal, showed the highest resistance to ampicillin (98%), and 94% of the isolates were multi-drug resistant [45]. On the contrary, European data from the Netherlands, France, and the UK, showed moderate resistance to tetracycline, streptomycin, ampicillin, and sulphonamides, with very low resistance in Sweden [46].

This difference in resistance can be attributed to the fact that the use of antimicrobials as growth promoters is restricted in European countries, whereas it is widely prevalent in the Asia region [11]. The high prevalence of resistance in Nepal could be due to the widespread misuse of antimicrobials among animals and poultry. Lack of strict rules and regulations may have contributed as well. A survey of the major distributors of veterinary medicines and feed supplements conducted in different cities of Nepal showed that tetracycline was the top antibiotic consumed in the veterinary sector [47].

According to the WHO, the main drivers of AMR include misuse and overuse of antimicrobials; lack of access to clean water and sanitation; poor access to quality, affordable medicines and diagnostics; lack of knowledge and awareness; and lack of enforcement of legislation [48]. Furthermore, a systematic review found poverty, lack of surveillance system, liberal rules, and regulations as AMR drivers from one health perspective in low and middle-income countries [49,50]. Moreover, our study found that there is a lack of protocol regarding the sales of antimicrobials and no legislation to regulate the production, distribution, sale, and prescription of antimicrobial agents in Nepal, promoting a widespread over-the-counter supply of drugs.

Interestingly, the interviewees perceived financial constraints as one of the factors that prevented the farmers from adhering to the prescribed duration of antimicrobials in our study. Issues such as lack of training among veterinary personnel and the selling of antimicrobials by unlicensed individuals were highlighted in our study. These factors may have contributed to the irrational sale of antimicrobials, whereas a systematic review reported indiscriminate use of antimicrobials, poor hygiene, insufficient staff training, and lack of proper management in livestock farms as leading causes of the high prevalence of *E. coli* isolates and its resistance to antimicrobials [51]. In Tanzania, self-prescription by drug shops and demand of farmers accounted for 59.7% of the total antibiotic sales [52]. A similar study conducted in Nepal also reported the prescription of drugs by non-professionals besides veterinarians as a serious problem [26]. Additionally, a review disclosed that over 70% of veterinary drugs sales were from para-professionals or retail outlets, whose staff usually have no veterinary training, and not prescribed by veterinary professionals in Nepal [24].

Furthermore, this study indicated that farmers fail to keep the minimum period of time between last dosage of antimicrobials and production of meat, due to ignorance. This might be due to a lack of awareness among farmers regarding AMR and its consequences. The failure to follow the withdrawal period leads to a low consumption of antimicrobials, which may increase the risk of microbial drug resistance and disruption of normal intestinal flora in humans [53]. In contrary, farmers of the broiler poultry farm in Kathmandu Valley stated that they acquired antibiotics through prescriptions and were aware and respected the withdrawal period. However, this information was not confirmed [54]. The high level of *E. coli* and its resistance to multiple drugs is alarming, as studies have already demonstrated the transmission of antibiotic-resistant bacteria from animals to humans [55,56]. As stated in the interviews, a surveillance system for AMR does not exist at present in Nepal, which seems to lack policies or surveillance systems to contain AMR. Even though several studies have been conducted to assess AMR in humans, animals, and the environment, these data have been underutilized, due to a lack of policy [26]. There is an urgent need for collaboration between the Department of Health Services (Ministry of Health and Population), Department of livestock services (Ministry of Livestock Development), and the Ministry of Health and Population, to implement a proper surveillance program [57].

## 5. Conclusions

A high level of multi-drug resistance was noted in the *E. coli* isolates from chicken cecal samples in the two wards of Dhulikhel municipality in Nepal. Overuse of antimicrobials, easy availability of falsified/substandard drugs, and a lack of awareness among farmers were the issues contributing to AMR. To overcome the high burden of AMR, the issues contributing to AMR have to be addressed by limiting the use of antimicrobials, by restricting over-the-counter sales, educating farmers on AMR, and establishing multi-sectoral coordination among stakeholders.

## Figures and Tables

**Table 1:** Characteristics of the distribution of the prevalence of *E. coli* and MDR in the *E. coli* isolated from chicken cecal samples from retail shops in two selected wards of the Dhulikhel municipality of Nepal, from September to December 2021.

Characteristics	Total	*E. coli* Present		MDR ^#^	
		N	(%)	N	(%)
Total	170	159	(93.5)	113	(71.1)
Types of meat sold					
Other * animal meat along with chicken	120	112	(93.3)	88	(78.6)
Only chicken	50	47	(94.0)	25	(53.2)
Type of shop					
Only sale	48	45	(93.8)	34	(75.6)
Slaughter house attached	122	114	(93.4)	79	(69.3)
Source of water					
Piped into dwelling	170	159	(93.5)	113	(71.1)
Ward					
Ward-2	48	45	(93.8)	34	(75.6)
Ward-6	122	114	(93.4)	79	(69.3)

* Buffaloes, Goats, Sheep, Pigs; ^#^ Multi-Drug Resistant.

**Table 2:** Pattern of resistance among *E. coli* isolated from chicken cecal samples in the Dhulikhel municipality of Nepal from September to December 2021 (N = 165).

Antimicrobial	Sensitive		Intermediate		Resistant	
	N	(%)	N	(%)	N	(%)
Cefotaxime	150	(90.9)	3	(1.8)	12	(7.3)
Ciprofloxacin	27	(16.4)	29	(17.6)	109	(66.1)
Ampicillin	47	(28.5)	19	(11.5)	99	(60.0)
Tetracycline	24	(14.5)	0	(0.0)	141	(85.5)
Chloramphenicol	102	(61.8)	12	(7.3)	21	(12.7)
Gentamicin	127	(77.0)	17	(10.3)	21	(12.7)
Cotrimoxazole	77	(46.7)	4	(2.4)	84	(50.9)

## Data Availability

The data that support the findings of this study are available from the corresponding author upon reasonable request.
